# Toll-Like Receptor 4 Inhibits Estradiol Secretion *via* NF-κB Signaling in Human Granulosa Cells

**DOI:** 10.3389/fendo.2021.629554

**Published:** 2021-03-12

**Authors:** Hai-Yun Guan, He-Xia Xia, Xiu-Ying Chen, Lu Wang, Zhi-Jing Tang, Wei Zhang

**Affiliations:** ^1^ Department of Reproductive Endocrinology, Obstetrics and Gynecology Hospital, Fudan University, Shanghai, China; ^2^ Shanghai Key Laboratory of Female Reproductive Endocrine Related Diseases, Shanghai, China

**Keywords:** *CYP19A1*, granulosa cell, steroidogenesis, Toll-like receptor 4, estradiol

## Abstract

Toll-like receptor 4 (TLR4) may play a critical role in regulating follicular development. Data are scarce on the role of TLR4 in the follicle. This study investigated the effects of TLR4 on steroidogenesis in human granulosa cells. Immunohistochemical analysis revealed stage-specific expression of TLR4 in the mouse ovarian cycle, and immunofluorescence showed TLR4 expression in the human granulosa-like tumor cell line (KGN). TLR4 agonist lipopolysaccharides (LPS) significantly inhibited follicular development and synthesis of estradiol (E2) in mice. In KGN cells, TLR4 activation significantly inhibited CYP19A1, FSHR and StAR, and TLR4 inhibition reversed these effects. TLR4 activation also inhibited forskolin-induced secretion of E2 by inhibiting CYP19A1, with no effect on progesterone. Further studies showed activation of p38, JNK and NF-κB signaling after TLR4 activation. Subsequent analyses showed that an NF-κB antagonist reversed the inhibitory effects on CYP19A1 expression and E2 secretion. Together, our results suggest that TLR4 activation may suppress CYP19A1 expression and E2 secretion *via* NF-κB signaling in human granulosa cells, with important implications for the regulation of ovarian pathophysiology.

## Introduction

In mammals, ovaries undergo cyclic variation both in terms of morphology and function, characterized by folliculogenesis, ovulation and luteinization. Granulosa cells (GCs) play a crucial role in these processes and are critical for supporting ovarian function and determining follicular fate (1). Importantly, GCs regulate their own proliferation, responsiveness to gonadotropin, apoptosis and steroidogenesis (2).

Steroidogenesis under the control of follicle stimulating hormone (FSH), luteinizing hormone (LH) and local regulators is an important physiological process, which influences the maturation and ovulation of follicles. In addition, steroidogenesis was reported to be associated with embryo quality. Higher levels of follicular E2 correlated well with successful fertilization following artificial reproduction treatment (3). E2 is the main steroid produced by GCs and is controlled by several factors, among which FSH plays a central role (4). FSH protects GCs from oxidative injury, also rescues GCs from apoptosis and dominant follicle atresia (5). FSH induces the production of E2 *via* FSHR-cAMP-dependent signaling to induce the transcription of the CYP19A1 gene (6). This gene encodes the cytochrome P450 enzyme aromatase, which convert androgens to estrogens (7). Progesterone is an essential reproductive hormone that is well-known to be produced by GCs immediately prior to ovulation (8). In addition, progesterone synthesis was found to be promoted by FSH from human GCs without luteinization (9); however, premature increase of progesterone before ovulation trigger may reduce pregnancy rates in stimulated *in vitro* fertilization cycles (10). Therefore, steroidogenesis plays a critical role in follicular development, ovulation and luteinization, and abnormal steroidogenesis may lead to decreased follicle survival, ovulation rates and fertility.

Toll-like receptors (TLRs) are highly conserved proteins of the innate immune system that detect various pathogen-associated molecular patterns and play a role in the subsequent immune response (11). Previous studies have evaluated the expression of TLRs in GCs from human and bovine ovaries (12, 13). Moreover, TLR signaling pathways were found to be induced in cumulus-oocyte complex samples collected from preovulatory follicles (13). Therefore, TLRs may play an important role in regulating follicular development and ovulation. TLR4 is a member of TLRs and is widely expressed on a variety of cell types in addition to immune cells. Ligand binding to TLR4 leads to the activation of the myeloid differentiation primary response 88 and TIR-domain-containing adapter-inducing interferon-β pathways, which causes the activation of several transcription factors such as nuclear factor κB (NF-κB) and interferon response factor 3, thereby promoting the release of proinflammatory cytokines (14). Previous studies analyzed the role of TLR4 in immunosurveillance, with scarce data evaluating the influence on local cell populations. Whilst the expression of TLR4 in human GCs has been revealed, the influence on GC function remains unknown. Therefore, we aimed to explore the role of TLR4 in steroidogenesis in human ovarian GCs.

## Materials and Methods

### Animals

Immature (3-week-old) female C57BL/6 mice were obtained from the SLAC Company (Shanghai, China). This study was approved by the experimental animal ethics committee of Fudan University and all experiments were performed under the guidelines of the animal care regulations of Fudan University. All mice were housed under controlled conditions on a 12h light/12h dark cycle and had free access to food and water. All agents were injected intraperitoneally. Mice were randomly divided into six groups as follows:

- Blank group (n=6): mice were injected with 100 μl PBS and sacrificed 48 h later. Ovaries were collected immediately for further analysis.
- Pregnant mare serum gonadotropin (PMSG) 48 h group (n=6): mice were injected with 5 IU PMSG (Easycheck, Nanjing, China) and were sacrificed 48 h later.
- Human chorionic gonadotropin (hCG) 2 h group (n=6): mice were injected with 5 IU PMSG and 48 h later, mice were injected with 10 IU hCG (Easycheck, Nanjing, China) to induce ovulation and luteum formation. They were sacrificed 2 h later.
- hCG 12 h group (n=6): mice were injected with 10 IU hCG following PMSG treatment as described above and sacrificed 12 h later.
- Control group (n=12): mice were treated with 5 IU PMSG along with 100 μl PBS (for 2 consecutive days). And mice (n=6) were sacrificed 48 h after PMSG treatment. Serum and ovaries were collected immediately for further study. Another 6 mice were treated with 10 IU hCG. After 15h, cumulus-oocyte complexes were released from the oviducts and digested with hyaluronidase (Sigma, St. Louis, USA), and the number of oocytes was counted.
- LPS group (n=12): mice were treated with 5 IU PMSG alongside 5mg/kg LPS (for 2 consecutive days). And mice (n=6) were sacrificed 48 h after PMSG treatment. Serum and ovaries were collected for further study. Another 6 mice were treated with 10 IU hCG. After 15 h, oocytes were collected and counted as described previously.

### Cell Culture

KGN, a steroidogenic human granulosa tumor cell line expressing CYP19A1 and functional FSHR, was purchased from Cellcook. Ltd. (Guangzhou, China). STR profiling was performed to ensure authenticity. Cells were cultured in DMEM/F12 without phenol red supplemented with 10% charcoal-treated fetal bovine serum. To maintain their unique functional qualities, cells were only used for experiments between passage 4 and passage 9. Lipopolysaccharides (LPS,1 μg/mL, Sigma, St. Louis, USA) was used as the agonist of TLR4 based on previous research (15). To investigate the mechanisms, cells were pretreated for 1 h with or without antagonists of specific pathways (TLR4 inhibitor TAK-242 (10 μM, MCE, New Jersey, USA), NF-κB signaling inhibitor JSH-23 (30 μM, MCE, New Jersey, USA), p38 MAPK signaling inhibitor SB 203580 (10 μM, MCE, New Jersey, USA) or JNK signaling inhibitor SP600125 (10 μM, MCE, New Jersey, USA)), and treated with LPS for 24 h. The antagonists are known to be specific in their effects at the concentrations listed based on previous reports (15–17). Recombinant follicle stimulating hormone (rFSH,1 IU, Merck-Serono, Geneva, Switzerland) and forskolin (FSK,1μM, Beyotime, Shanghai, China) were used to stimulate steroidogenesis.

### Immunohistochemical Analysis

Immunohistochemical analysis was performed to analyze the localization of TLR4 in the ovaries. Ovaries were immersed in 4% paraformaldehyde for fixation, and then embedded in paraffin and cut into 4 μm sections for histological analysis. The slides were incubated in citrate buffer (pH 6.0) and heated at 98°C for 30 mins for antigen retrieval. After recovering to room temperature, the slides were incubated with 3% H_2_O_2_ for 10 mins to inhibit peroxidase activity followed by blocking with 10% goat serum for 1 h. Slides were then incubated with primary antibody against TLR4 (GB11519, 1:500) overnight at 4°C. For the negative control, IgG from rabbit serum was used instead of primary antibodies. The slides were incubated with horseradish peroxidase (HRP)-conjugated goat anti-rabbit antibody (GB23303, 1:500) for 1h at room temperature. Immunoreactive signals were observed using 3,3-diaminobenzidine. And the slides were counterstained with hematoxylin. Images of the stained slides were obtained using an Olympus BX53 microscope (Olympus, Tokyo, Japan).

### Immunofluorescence

KGN cells were seeded on cover slips in 6-well plates and cultured for 24 h. Cells were then fixed with 4% paraformaldehyde and permeabilized with 0.1% Triton X-100 on ice, followed by blocking with goat serum. Then, the cells were incubated with TLR4 antibody (GB11519, 1:1000) overnight at 4°C. The negative control was incubated with rabbit IgG isotype control (A7016, 1:1000). The next day, cells were incubated with FITC-conjugated goat anti-rabbit secondary antibodies (1:500, A0562) for 1 h at room temperature. Then nuclei were stained with DAPI (1.5 μM, Beyotime, Shanghai, China) for 10 mins. Images were obtained using Nikon fluorescence microscopy (Nikon, Tokyo, Japan).

### Histology

Ovaries were fixed with 4% paraformaldehyde and embedded in paraffin, serially sectioned in the longitudinal plane with a thickness of 4μm. Every 15^th^ section was stained with hematoxylin and eosin (HE). The numbers of primordial, primary, secondary and antral follicles were counted. Follicles with a single layer of flattened GCs were classified as primordial follicles. Follicles with a single layer of cuboidal GCs were counted as primary follicles. Follicles with multiple layers of cuboidal GCs were classified as secondary follicles. Follicles with two or more layers of GCs and A fluid-filled antral space were counted as antral follicles (18).

### Quantitative Real-Time PCR

Total RNA was isolated with TRIZOL regent (Invitrogen, Carlsbad, USA), and cDNA was synthesized with PrimeScript™ RT Reagent Kit (Takara, Otsu, Japan). The respective primers are shown in **Table 1**. Quantitative real-time PCR (qRT-PCR) was carried out using the TB Green™ Premix Ex Taq™ II Kit (Takara, Otsu, Japan) in the ABI 7900 real-time PCR system (Applied Biosystems Inc., Foster City, USA). The 2^−ΔΔCt^ method was used to evaluate the fold change at the transcriptional level.

**Table 1:** Primers used for qRT-PCR.

Gene	Forward primers	Reverse primers
*GAPDH*	CTGGGCTACACTGAGCACC	AAGTGGTCGTTGAGGGCAATG
*CYP19A1*	ACTACAACCGGGTATATGGAGAA	TCGAGAGCTGTAATGATTGTGC
*FSHR*	TCTGTCACTGCTCTAACAGGG	TGCACCTTTTTGGATGACTCG
*StAR*	GGGAGTGGAACCCCAATGTC	CCAGCTCGTGAGTAATGAATGT
*3β* ***-*** *HSD*	CACATGGCCCGCTCCATAC	GTGCCGCCGTTTTTCAGATTC
*CYP11A1*	GCTTTGCCTTTGAGTCCATCA	CTCGGGGTTCACTACTTCCTC

### Western Blot Analysis

Western blot was performed as previously described (19). Briefly, protein samples were separated by 12% SDS-PAGE gels (Beyotime, Shanghai, China) and then transferred to PVDF membrane. Membranes were blocked with 5% skimmed milk for 1 h at room temperature and incubated at 4°C overnight with the following primary antibodies: Anti-GAPDH (AF1186, 1:3000), Anti-CYP19A1 (ab124776, 1:5000), Anti-StAR (12225-1-AP, 1:500), Anti-FSHR (22665-1-AP, 1:1000), Anti-3β-HSD (sc-100466, 1:1000), Anti-NF-κB p65 (AN365, 1:500), Anti-Phospho-NF-κB p65(Ser536) (AN371, 1:500), Anti-JNK (bs-10562R, 1:500), Anti-Phospho-JNK (bs-1640R, 1:500), Anti-p38 MAPK (bs-0637R, 1:500) and Anti-Phospho-p38 MAPK (bs-5477R, 1:500). The membranes were washed in TBST three times and then incubated with HRP-conjugated goat anti-rabbit antibody (GB23303, 1:3000). The bands were detected with enhanced chemiluminescence (Millipore, Danvers, MA, USA) and analyzed using Image J Software. The ratios of phosphorylated and normal protein to GAPDH were calculated to determine the relative phosphorylation of certain proteins. Quantitative analysis of images was performed using Image J software.

### Enzyme-Linked Immunosorbent Assay

KGN cells were seeded in 24-well plates at a density of 4×10^4^ cells per well in 1 ml of DMEM/F12 without red phenol and with charcoaled-treated 10% fetal bovine serum. Cells were cultured with rFSH (1IU, Merck-Serono, Geneva, Switzerland) or FSK (1 μM, Beyotime, Shanghai, China) in the presence and absence of LPS or specific inhibitors for 48 h. To detect E2 secretion, testosterone (10 ng/ml) was added into the medium as a substrate for E2 synthesis. After 48 h, cell supernatant was collected to detect E2 with the estradiol ELISA kit (FR E-2000, LDN, Nordhorn, Germany) according to the manufacturer’s instructions. Medium from the cells treated for 48 h with rFSH or FSK in the presence and absence of LPS was collected for detecting progesterone using a progesterone ELISA kit (JL15420, Jianglai Biology, China). The levels of serum E2 were detected with the mouse estradiol ELISA kit (JL11232, Jianglai Biology, China) according to the manufacturer’s instructions.

### Statistical Analyses

Data were obtained from at least three independent experiments and expressed as mean ± S.D. Student t-test, one-way analysis of variance (ANOVA) followed by Tukey’s multiple comparisons test or Dunnett’s multiple comparisons and Kruskal-Wallis test followed by Dun’s multiple comparisons were used to analyze the differences between groups. The threshold for statistical significance was P<0.05.

## Results

### TLR4 Expression and Localization in Mouse Ovaries and Human Granulosa Cells

**Figure 1:**
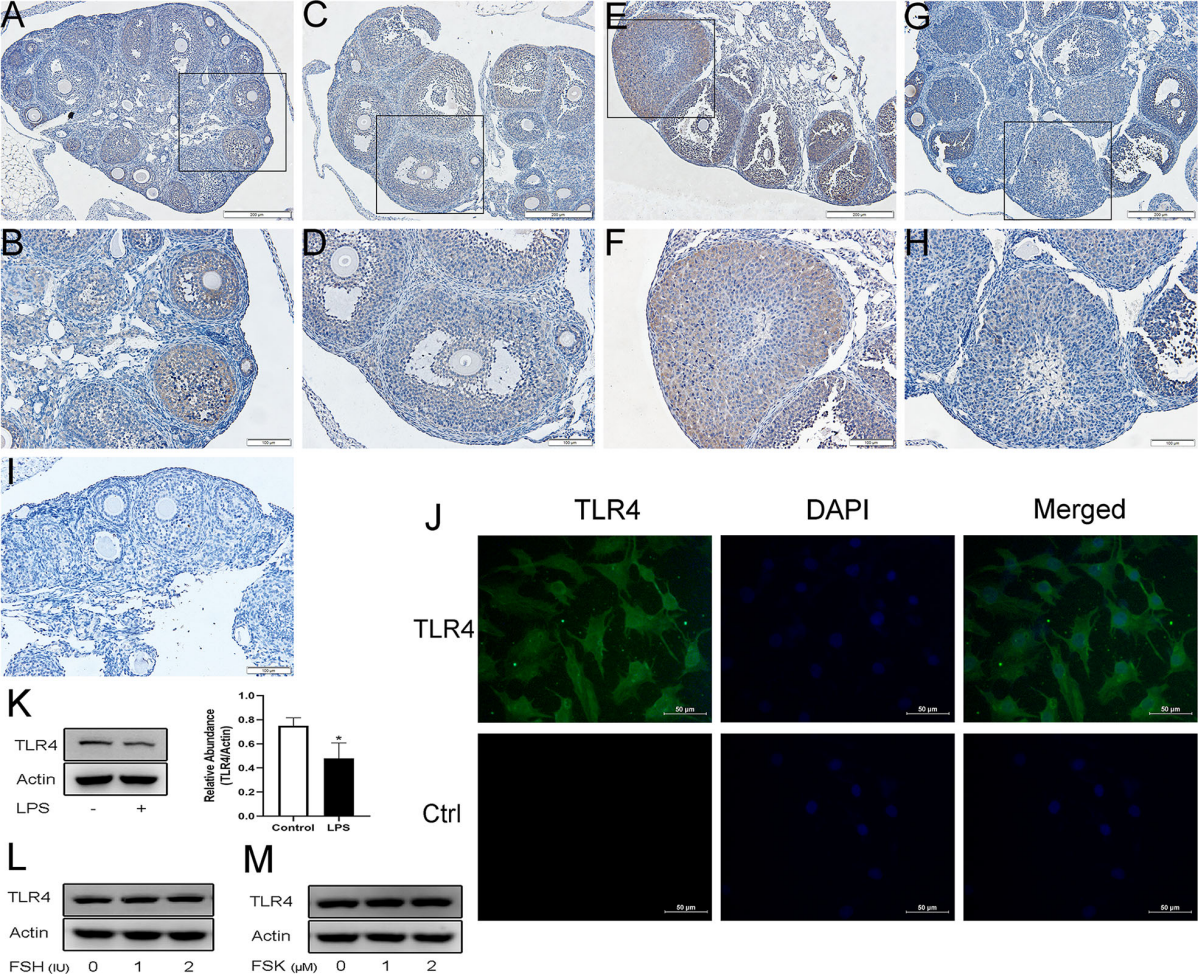
Expression and localization of TLR4 in mouse ovaries and KGN cells. Representative immunostaining images of mouse ovaries at different stages of follicle development are displayed. TLR4 is predominantly observed in GCs and the corpus luteum. **(A, B)** Immature mice treated with PBS. **(C, D)** Immature mice treated with PMSG for 48 h. **(E, F)** Immature mice treated with PMSG for 48 h and then hCG for 2 h. **(G, H)** Immature mice treated with PMSG for 48 h and then hCG for 12 h. **(I)** Negative control. **(J)** Identification of TLR4 protein (visualized in green FITC) in KGN cells using fluorescent immunocytochemistry. Control (Ctrl) refers to the negative control. **(K)** KGN cells were treated with 1μg/ml LPS for 24 h, western blot analysis of TLR4 expression. **(L)** KGN cells were treated with FSH at various concentrations for 24 h, western blot analysis of TLR4 expression. **(M)** KGN cells were treated with FSK at various concentrations for 24 h, western blot analysis of TLR4 expression. *P < 0.05, Scale bar = 200 μm **(A, C, E, G)**, scale bar = 100 μm **(B, D, F, H, I)**, scale bar = 50 μm **(J)**.

Immunohistochemistry confirmed that within the ovary TLR4 is predominantly located in GCs and luteal cells. As shown in **Figures 1A–H**, TLR4 was detectable in GCs at different stages of follicles. It was relatively weakly expressed in GCs from mature follicles (**Figures 1C–D**), whereas it significantly increased both in GCs and luteal cells in response to hCG treatment (**Figures 1E–F**). In contrast, TLR4 was significantly decreased in the corpus luteum in the hCG 12 h group (**Figures 1G–H**). These findings indicate that TLR4 shows stage-specific expression in GCs and the corpus luteum, which may suggest a role for TLR4 in regulating follicular development and luteinization. Previous studies have shown the expression of TLR4 in human GCs (20). We further examined TLR4 localization in KGN cells, As shown in **Figure 1J**, green immunofluorescence confirmed the expression of TLR4 in KGN cells. In addition, we further examined the effects of TLR4 agonist LPS, FSH and FSK on TLR4 expression in KGN cells. As evidenced in **Figure 1K**, the protein levels of TLR4 decreased after treatment of LPS for 24 h (P=0.0317). No obvious changes were observed in TLR4 expression after the stimulation of FSH and FSK for 24 h (**Figures 1L–M**).

### LPS Inhibited Follicular Development in Mice

**Figure 2:**
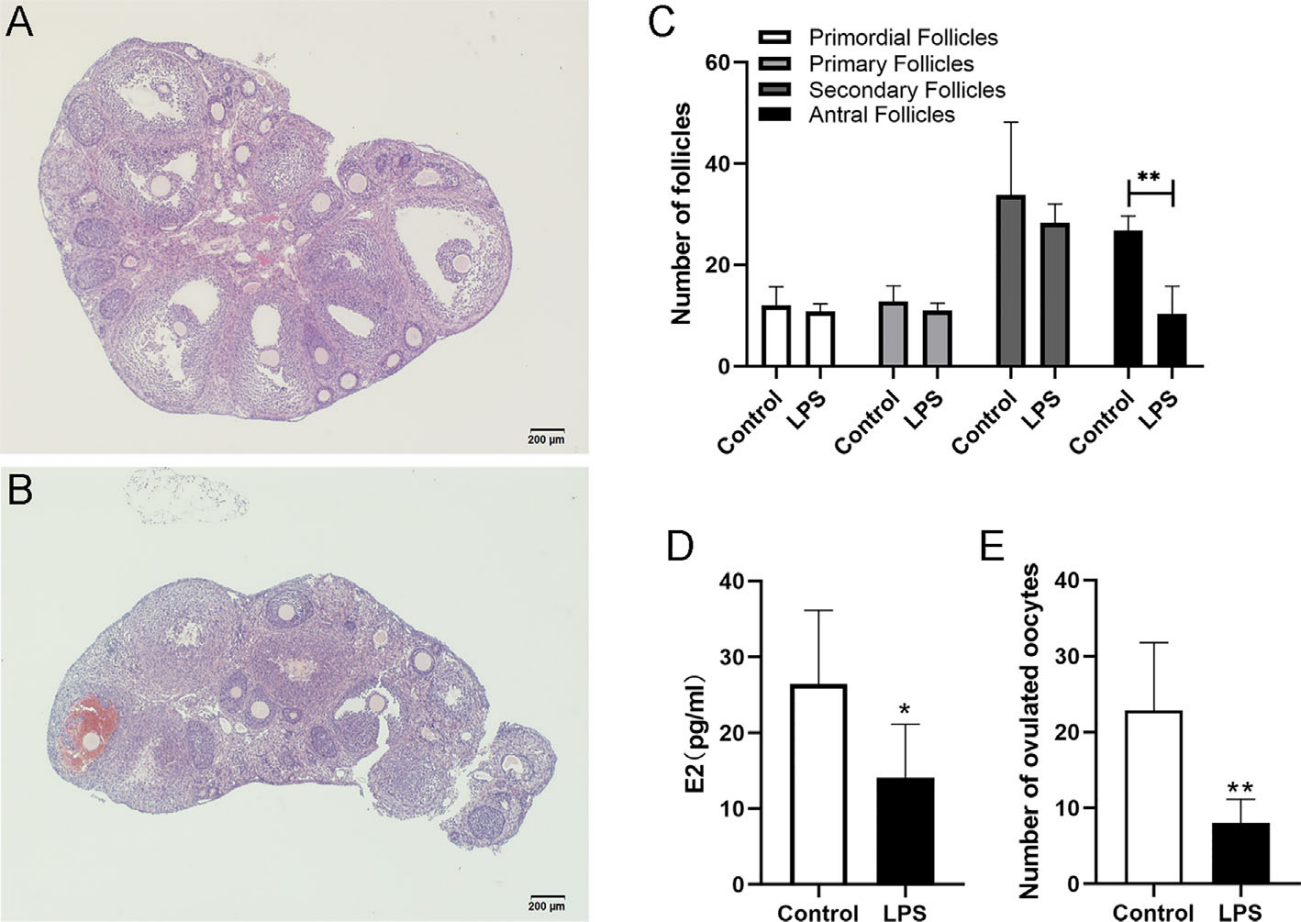
LPS inhibited follicular development in mice. Immature female mice received PMSG with LPS or PBS. Ovaries were fixed for histology analysis 48 h later. **(A)** Representative HE staining of ovary from the control group. **(B)** Representative HE staining of ovary from the LPS group. **(C)** Numbers of follicles at different stages in mice of the control and LPS group. **(D)** Serum E2 levels of mice at 48 h after PMSG treatment. **(E)** Immature female mice received PMSG with LPS or PBS, followed by hCG 48 h later. Oocytes were obtained 15 h post hCG stimulation. Numbers of ovulated oocytes were counted. All data are means ± S.D. n=6. Student t-test was used to analyze the differences. *P <0.05, **P <0.01. Scale bar = 200μm **(A, B)**.

To examine the effects of TLR4 activation on follicular development, TLR4 agonist LPS was co-administered with PMSG. In this way, the mice were exposed to LPS during the growth period of follicles. Results showed that numerous large antral follicles are observed in the control group after 48 h treatment of PMSG (**Figure 2A**). Correspondingly, few antral follicles were observed in the LPS group (**Figure 2B**). As shown in **Figures 2C–D**, the LPS group had significantly fewer antral follicles (P=0.0028) and E2 levels (P=0.0113) than the control group, but the two groups did not differ significantly in numbers of pre-antral follicles. For 15 h after hCG treatment, the ovulated oocytes were collected and counted. And we observed that the number of ovulated oocytes is significantly decreased in the LPS group (P=0.0034). These results suggest that TLR4 activation inhibited follicular development in mice.

### TLR4 Signaling Regulated Steroidogenic Genes in KGN Cells

**Figure 3:**
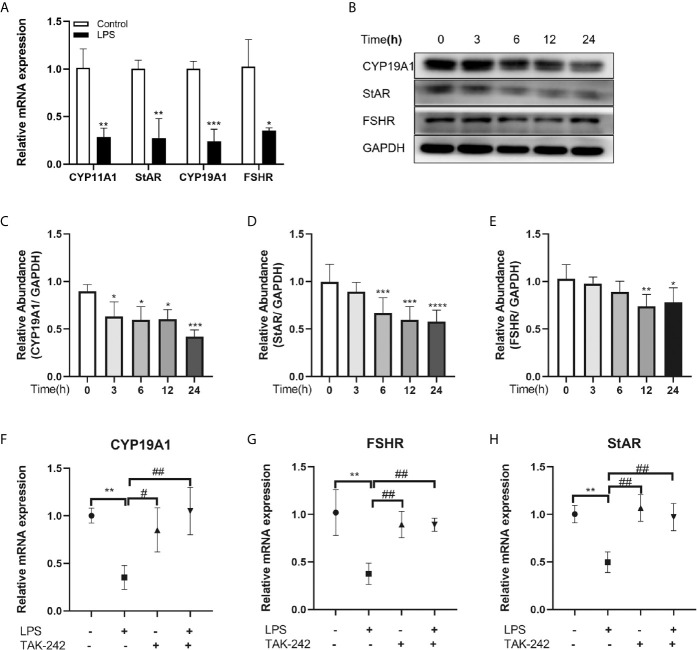
TLR4 signaling regulated steroidogenic genes in KGN cells. **(A)** Effects of TLR4 activation on transcription of key steroidogenic genes, including CYP11A1, StAR, CYP19A1 and FSHR. **(B)** Western blot analysis of CYP19A1, StAR and FSHR expression in KGN cells treated with TLR4 agonist LPS over time (0, 3, 6, 12, 24 h). **(C–E)** Quantitative analysis of immunoblot bands. **(F-H)** TAK-242, a TLR4 inhibitor, reversed the inhibition of LPS on the transcription of CYP19A1, FSHR and StAR. All data are means ± S.D. n=3. Student t-test and one way ANOVA followed by Tukey’s multiple comparisons test were used to analyze the differences. *P < 0.05, **P < 0.01 and ***P < 0.001 vs control cells. ^#^P < 0.05 and ^##^P < 0.01 vs LPS group.

Steroidogenesis is essential for follicular development and selection, so we examined whether the activation of TLR4 signaling is involved in the regulation of steroidogenic genes, levels of CYP19A1, StAR, CYP11A1 and FSHR, which are the key genes regulating E2 and progesterone in GCs, were investigated by qRT-PCR. As shown in **Figure 3A**, the TLR4 agonist LPS dramatically reduced the transcription levels of CYP11A1, CYP19A1, StAR and FSHR, respectively (P=0.0044, P=0.0009, P=0.0047, P=0.0149). Subsequently, cells were treated with LPS for different periods of time. Time-dependent changes in protein levels of CYP19A1, StAR and FSHR are shown in **Figures 3B–E**. Levels of CYP19A1, StAR and FSHR were markedly reduced after treatment with LPS. In addition, the TLR4 inhibitor TAK-242 reversed the inhibitory effects of LPS on CYP19A1, FSHR and StAR (P=0.0044, P=0.0084, and P=0.0037, respectively; **Figures 3F–H**). These results indicate that TLR4 signaling is involved in the regulation of steroidogenic genes.

### Effects of TLR4 Activation on rFSH-Stimulated Estradiol and Progesterone Production in KGN Cells

**Figure 4:**
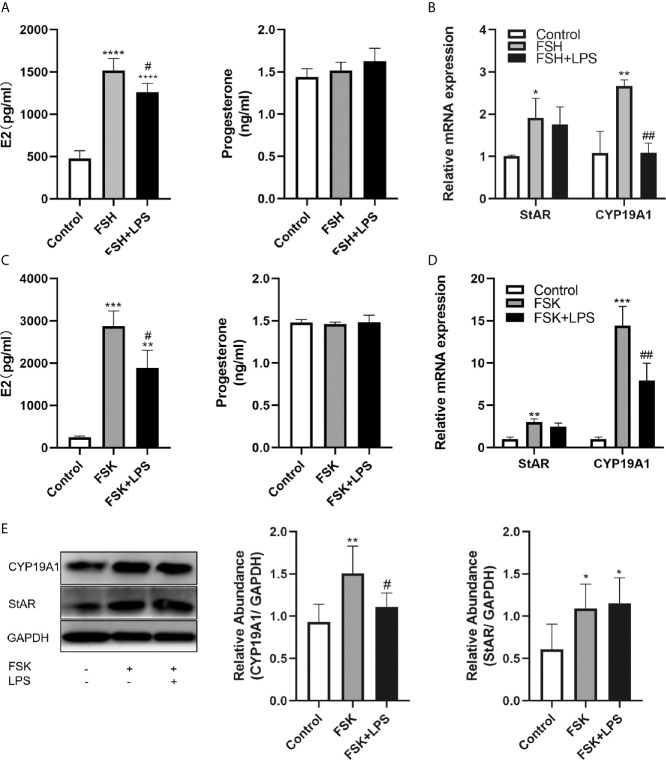
Effects of TLR4 activation on estradiol and progesterone secretion in KGN cells. **(A)** rFSH-induced secretion of E2 was reduced when co-incubated with LPS, while there was no difference in progesterone secretion. **(B)** Incubation of rFSH with LPS reduced rFSH-induced expression of CYP19A1 mRNA, with no effect on StAR mRNA. **(C)** FSK-induced secretion of E2 was also reduced when incubated with LPS, and there was no difference in progesterone secretion. **(D)** Incubation of LPS with FSK reduced FSK-induced transcription of CYP19A1, with no effect on StAR mRNA. **(E)** Incubation of LPS with FSK reduced FSK-induced protein level of CYP19A1, while there was no difference in StAR. All data are means ± S.D. n=3. One-way ANOVA followed by Tukey’s multiple comparisons test were performed to analyze the differences. *P<0.05, **P < 0.01, ***P < 0.001 and ****P<0.0001 vs control group. ^#^P < 0.05 and ^##^P < 0.01 vs FSH or FSK groups.

We further evaluated the effect of TLR4 activation on rFSH-stimulated steroid hormone production. Results showed that 48 h incubation with rFSH induced a 3-fold increase in E2 expression (P<0.0001) and no significant increase in progesterone levels (**Figure 4A**). However, TLR4 activation significantly inhibited the rFSH-induced production of E2 (P=0.0305). There was a rising trend for progesterone production among incubation of rFSH with LPS compared to rFSH alone, but this trend was not significant (**Figure 4A**).

### TLR4 Activation Suppressed Estradiol Secretion Through Inhibition of CYP19A1

To explore the potential causes underlying the decreased E2 secretion, we examined the expression changes of the steroidogenic enzymes, StAR and CYP19A1, which are key regulators of E2 and progesterone production by GCs. Results showed that incubation with rFSH for 24 h led to significant increases in StAR and CYP19A1 mRNA expression (P=0.0455 and P=0.0027, respectively). However, the activation of TLR4 signaling significantly decreased rFSH-induced CYP19A1 mRNA expression (P=0.0028). In addition, StAR mRNA expression induced by rFSH was not affected (**Figure 4B**). Considering the inhibition of TLR4 activation on FSHR, we used the adenylate cyclase activator FSK to confirm the inhibitory effect of TLR4 activation on CYP19A1. As shown in **Figure 4C**, FSK led to 11-fold increase in E2 expression (P=0.0001) and no significant increase in progesterone, and TLR4 activation significantly inhibited the FSK-induced production of E2 (P=0.0214). Similar to rFSH, FSK facilitated the transcription of CYP19A1 and StAR (P=0.0002 and P=0.0007, respectively). TLR4 activation also decreased the transcription (P=0.0098, **Figure 4D**) and protein level (P=0.0298, **Figure 4E**) of CYP19A1 induced by FSK, with no inhibitory effect on StAR. These results indicate that TLR4 activation suppresses E2 secretion through inhibition of CYP19A1.

### NF-κB Signaling Mediated LPS-Induced Inhibition on CYP19A1

**Figure 5:**
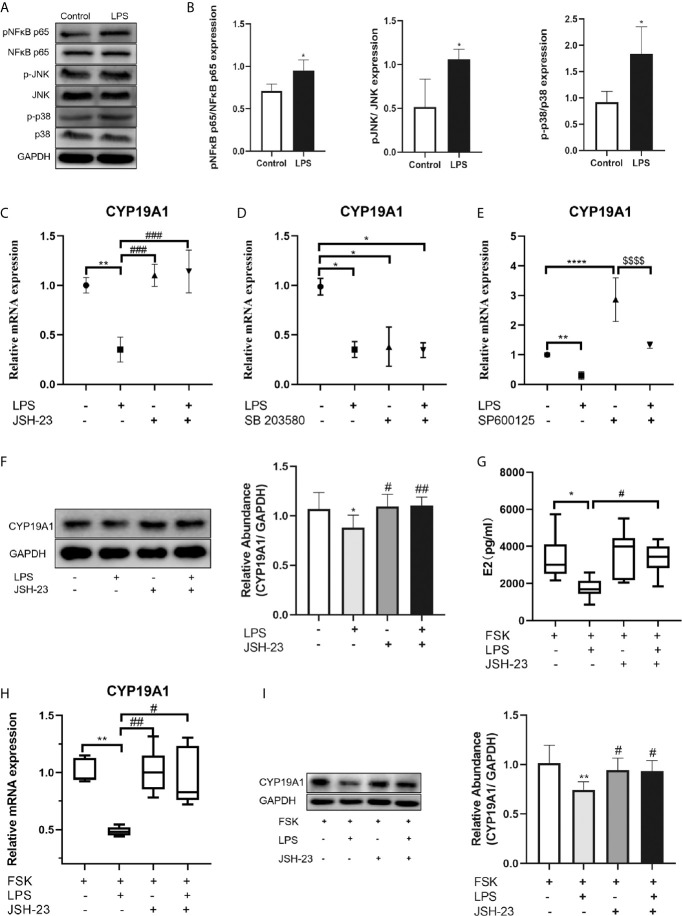
NF-κB signaling mediated the inhibition of TLR4 activation on CYP19A1 and E2 secretion. **(A)** KGN cells were treated with or without LPS for 3h. LPS induced phosphorylation of NF-κB p65, JNK and p38 MAPK in KGN cells. **(B)** The relative phosphorylation of NF-κB p65, JNK and p38 MAPK were analyzed (n=4). **(C–F)** The NF-κB signaling inhibitor JSH-23, p38 MAPK signaling inhibitor SB 203580, and JNK signaling inhibitor SP600125 were incubated either alone or with LPS for 24 h. **(C)** JSH-23 reversed the inhibition of LPS on CYP19A1 mRNA (n=3). **(D)** When incubated alone, SB 203580 markedly inhibited CYP19A1 mRNA expression (n=5). **(E)** SP600125 significantly promoted the expression of CYP19A1 mRNA and co-stimulation of LPS with SP600125 inhibited the facilitatory effect of SP600125 on CYP19A1 mRNA level (n=3). **(F)** Coincubation of JSH-23 with LPS for 24 h reversed the inhibition on the protein level of CYP19A1 (n=7). **(G)** Coincubation of JSH-23 with FSK and LPS for 48 h rescued the inhibition of LPS on FSK-induced E2 secretion (n=7). **(H)** Coincubation of JSH-23 with FSK and LPS for 24 h reversed the inhibition of LPS on FSK-induced expression of CYP19A1 mRNA (n=6). **(I)** Coincubation of JSH-23 with FSK and LPS for 24 h rescued the inhibition of LPS on the protein level of CYP19A1 (n=6). The results are expressed as means ± S.D. Student t-test, one-way ANOVA followed by Tukey’s multiple comparisons test and Kruskal-Wallis test followed by Dun’s multiple comparisons were used to analyze the differences. *P<0.05, **P < 0.01, ***P < 0.001 and ****P<0.0001 vs control or FSK group. ^#^P < 0.05, ^##^P<0.01 and ^###^P < 0.001 vs LPS or FSK+ LPS group. ^$$$$^P < 0.0001 vs SP600125 group.

The downstream signaling of the TLR4 pathway includes the MAPK and NF-κB pathways. To determine the downstream signaling cascade in KGN cells, we examined the activation of NF-κB, p38 and JNK after LPS treatment by Western blotting. The phosphorylation of NF-κB p65, p38 MAPK and JNK were enhanced after LPS treatment (P=0.019, P=0.016, and P=0.019, respectively; **Figures 5A–B**), indicating that LPS could activate NF-κB signaling pathway in KGN cells, as well as the p38 MAPK and JNK signaling pathways. We next investigated the potential mechanisms by which TLR4 signaling inhibits CYP19A1. We explored NF-κB, p38 MAPK and JNK signaling using specific inhibitors. The inhibitory effect on CYP19A1 mRNA induced by LPS stimulation was markedly reversed by pretreatment with the NF κB inhibitor JSH-23 (P=0.0004, **Figure 5C**). Similar results were observed in protein levels of CYP19A1 by pretreatment with JSH-23 (P=0.0095, **Figure 5F**). When incubated alone, p38 MAPK inhibitor SB 203580 substantially inhibited the transcription of CYP19A1 (P=0.0175, **Figure 5D**), while the JNK inhibitor SP600125 significantly promoted the transcription of CYP19A1 (P<0.0001, **Figure 5E**). Coincubation of SP600125 with LPS inhibited the facilitatory effect of SP600125 on CYP19A1 mRNA levels (P<0.0001). These results demonstrate that NF-κB signaling plays a key role in the inhibitory effects of TLR4 activation on CYP19A1 expression. Moreover, JSH-23 dramatically reversed the inhibitory effects of LPS on FSK-induced secretion of E2 (P=0.0486, **Figure 5G**), as well as transcription and protein levels of CYP19A1 (P=0.0429, P=0.0473, **Figures 5H–I**). Taken together, these results suggest that NF-κB signaling mediates the inhibition of TLR4 activation on CYP19A1 and E2 secretion.

## Discussion

Earlier studies showed that TLR4 is expressed in human cumulus cells. Recently, a study highlighted TLR4 expression in human primordial and primary follicles, with apparent staining in GCs (21). Consistent with these reports, we observed TLR4 expression in mouse GCs at different stages. In addition, the TLR4 agonist LPS can stimulate TLR4 target genes in ovaries *in vitro*, as well as cumulus cells from cumulus-oocyte complexes (22). These findings indicate that TLR4 signaling in human GCs is complete and functional. However, previous studies looked more closely at role of TLR4 in immunosurveillance, with scarce data evaluating the influence on functionality of local cell populations. Moreover, the influence of TLR4 activation on steroidogenesis in human GCs is less well understood.

**Figure 6:**
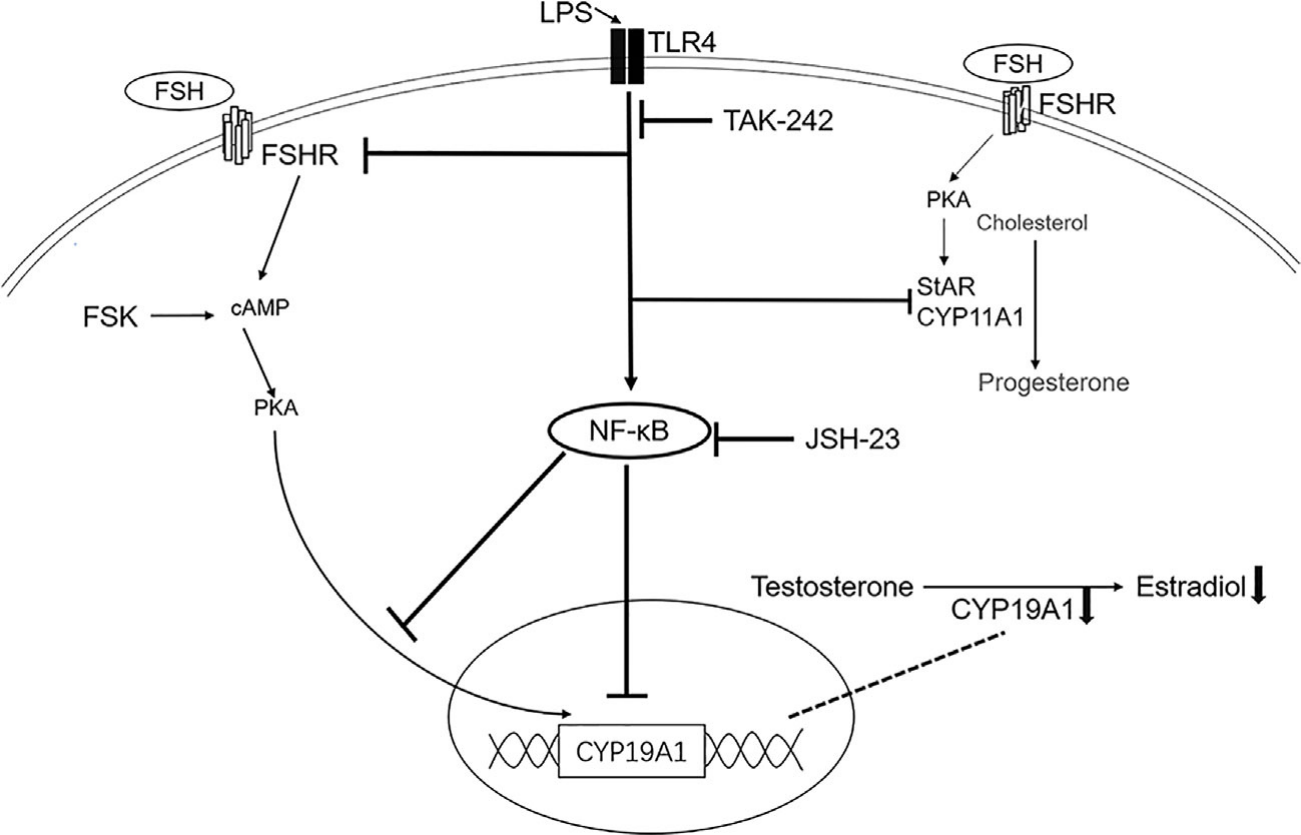
Schematic presentation of TLR4 activation in steroidogenesis in human granulosa cells. The TLR4 agonist LPS inhibited the expression of CYP11A1, StAR, FSHR and CYP19A1. The TLR4 inhibitor TAK-242 reversed the inhibition of LPS on CYP19A1, FSHR and StAR. TLR4 activation also inhibited FSH- and FSK-induced expression of CYP19A1 and estradiol secretion, with no effect on progesterone secretion. Importantly, TLR4 activation led to the activation of NF-κB signaling. The NF-κB inhibitor, JSH-23, rescued the inhibition on both basal and FSK induced expression of CYP19A1, as well as FSK-induced estradiol secretion.

We present evidence that TLR4 activation suppresses follicular development in mouse ovary. And in the human GCs, the basal expression of CYP19A1 is significantly inhibited. Indeed, some studies in mammals found that LPS decreases CYP19A1 expression *in vitro* (12, 23, 24). In addition, TLR4 was involved in the downregulation of CYP19A1 (23). Our data are in accordance with these findings, further confirming the inhibitory effect of TLR4 on CYP19A1. Moreover, we illustrated for the first time that TLR4 activation also inhibits rFSH- and FSK-induced expression of CYP19A1, as well as E2 secretion in human GCs. In addition, TLR4 signaling may suppress FSH activity by inhibiting the expression of FSHR. Though FSH is a major survival factor for antral follicles and GCs, activation of TLR4 signaling compromised the ability of rFSH to stimulate CYP19A1 and consequent E2 production. Thus, the development of follicles can be restricted. Furthermore, TLR4 activation led to the activation of NF-κB, JNK and p38 MAPK signaling. According to our data, although JNK and p38 MAPK may be involved in the regulation of CYP19A1, NF-κB predominantly inhibited TLR4 activation of CYP19A1 and E2 secretion. Collectively, these data suggest that TLR4 signaling impacts E2 production and FSH response in GCs (**Figure 6**).

Our study suggests a new role for TLR4 in ovulation. Ovulation is well known to be linked to the inflammatory response, with many genes associated with immune surveillance induced in cumulus cells, including TLR4 signaling (22). TLR4 signaling is thought to play critical role in surveillance during ovulation (25). We did observe the enhancement of TLR4 expression after ovulation triggering, further suggesting a role of TLR4 in ovulation. Importantly, after the ovulatory LH surge, the expression of CYP19A1 is rapidly suppressed, and GCs shift from estrogen to progesterone synthesis, which is crucial for ovulation and following luteinization (26). Though LPS is not part of normal mammalian physiology, TLR4 can also be activated by numerous endogenous ligands. Currently, the role of TLR4 in ovulation is not well understood. And the ligand which may regulate the TLR4 signaling in the process is uncertain. We speculate that TLR4 is likely to play a role in the regulation of steroidogenesis in ovulatory GCs, but further studies are needed to verify this speculation.

Our study also implies that TLR4 signaling may be involved in abnormal follicular development and selection in some inflammatory diseases. It is well known that the production of estrogen is essential for follicular development and selection. A previous study demonstrated that estrogen promotes survival and growth of follicles at the preantral to early antral stage (27), and disruption of these processes may drive follicular atresia. LPS is a major component of Gram-negative bacteria, and the bacteria is closely associated with pelvic inflammatory disease (PID), which is a common gynecological disease. And TLR4 can recognize Gram-negative bacteria through LPS. Apart from PID, LPS was also found elevated in obese women with PCOS (28). Abnormal elevated LPS in circulation or the microenvironment can activate the TLR4 signaling in GCs. Our data showed that TLR4 is detectable in GCs at different stages of follicles. We also present evidence that numbers of pre-antral follicles are unchanged, but follicular development can be disturbed after LPS stimulation. On the other hand, upregulated TLR4 and its downstream targets can also cause TLR4 hyperactivation, leading to detrimental effects (12). A previous report revealed that GCs from women with polycystic ovary syndrome (PCOS) express higher levels of TLR4, which is associated with lower embryo quality (20). TLR4 can be upregulated in several cases. For example, our data showed increased expression of TLR4 after stimulation of hCG. Moreover, lipid challenge was found to upregulate TLR4 expression in mononuclear cell in PCOS patients (28). Therefore, upregulated expression of TLR4 and its downstream targets, as well as abnormally elevated LPS may inhibit the production of E2 in GCs and influence the development of follicles. Our findings indicate that hyperactivation of TLR4 signaling may be implicated in the abnormal follicular development observed in PCOS.

## Conclusions

Our data demonstrate for the first time that TLR4 activation inhibits follicular development in mice, suppresses basal and FSK-induced CYP19A1 expression, as well as E2 secretion in human GCs. In addition, TLR4 activation also compromised FSH activity by inhibiting the expression of FSHR. Furthermore, we showed the involvement of NF-κB signaling in mediating the inhibition on CYP19A1 and E2 secretion. Thus, TLR4 activation may affect reproductive capacity, which suggests a novel role of TLR4 signaling in ovarian pathophysiology.

## Data Availability Statement

The original contributions presented in the study are included in the article/supplementary material. Further inquiries can be directed to the corresponding author.

## Ethics Statement

The animal study was reviewed and approved by the experimental animal ethics committee of Fudan University.

## Author Contributions

H-YG, H-XX and WZ conceived and designed the study. H-YG, X-YC, LW, and Z-JT performed experiments and analysis. H-YG, H-XX, and LW wrote sections of the manuscript. All authors contributed to the article and approved the submitted version.

## Funding

This work was supported by National Natural Science Foundation of China (81771587).

## Conflict of Interest

The authors declare that the research was conducted in the absence of any commercial or financial relationships that could be construed as a potential conflict of interest.

## Abbreviations

KGN, human granulosa-like tumor cell line; GCs, Granulosa cells; TLR4, toll-like receptor 4; HRP, horseradish peroxidase; HE, hematoxylin and eosin; CYP19A1, Cytochrome P450 Family 19 Subfamily A Member 1; StAR, steroidogenic acute regulatory protein; CYP11A1, Cytochrome P450 Family 11 Subfamily a Member 1; E2, estradiol; FSHR, follicle stimulating hormone receptor; FSH, follicle stimulating hormone; LH, luteinizing hormone; TLRs, toll-like receptors; NF-κB, nuclear factor-kappa B; PMSG, pregnant mare serum gonadotropin; hCG, human chorionic gonadotropin; LPS, lipopolysaccharides; qRT-PCR, quantitative real-time PCR; rFSH, recombinant follicle stimulating hormone; FSK, forskolin; PCOS, polycystic ovary syndrome; PID, pelvic inflammatory disease.
